# Open-Design for a Smart Cover of a Night-Time Telescope for Day-Time Use

**DOI:** 10.3390/s21041138

**Published:** 2021-02-06

**Authors:** Raquel Cedazo, Alberto Brunete, Hugo R. Albarracin, Esteban Gonzalez

**Affiliations:** 1 Department of Electrical, Electronical and Automatic Control Engineering and Applied Physics, Escuela Técnica Superior de Ingeniería y Diseño Industrial, Universidad Politécnica de Madrid, 28012 Madrid, Spain; h.albarracin@alumnos.upm.es; 2 Centre for Automation and Robotics (CAR UPM-CSIC), Universidad Politécnica de Madrid, 28006 Madrid, Spain; alberto.brunete@upm.es; 3 Ontology Engineering Group (OEG), Universidad Politécnica de Madrid, 28660 Madrid, Spain; egonzalez@fi.upm.es

**Keywords:** astronomy, autonomous observatory, fabrication laboratory, low-cost components, mechatronics, open design, protection system, robotic telescopes, smart device

## Abstract

Robotic observatories are ideal infrastructures that can be remotely accessed by scientists, amateurs, and general public for research and education in Astronomy. Its robotization is a complex process for ensuring autonomy, safety, and coordination among all subsystems. Some observatories, such as Francisco Sanchez’s, are equipped with two types of telescopes: one for the night and one for the day. The night-time telescope must be protected from exposure to sunlight in order to use them in an automated way. For this purpose, this article proposes the design and construction of a smart cover that opens and closes according to the time of day. The mechatronic design covers the electronic, mechanical, and software programming, and it has been devised taking while taking the principles of open design, ease of reproduction, low-cost, and smart behaviour into account. The design has been parameterized, so that it can be adapted to telescopes of any size. The final prototype is lightweight, cost-effective, and can be built while using common 3D printing and PCB milling machines. The complete design is licensed under the GNU General Public License v3.0 and all the documentation, schematics, and software are available in public repositories, like Zenodo, GitHub, and Instructables.

## 1. Introduction

The astronomical observatories are complex infrastructures in privileged places, away from light pollution, in order to make observations in the best conditions. Consequently, their access is not easy, since they are far from inhabited areas. The invention of robotic observatories has been a major breakthrough for Astronomy and it has meant a big advance for scientists, who are able to telework from remote places or, even, in such unusual confinement situations as the one experienced with who are able to telework from COVID-19.

Robotization and automation are not trivial, but, with the evolution of control technology and communications, increasing modern observatories are being automated. There is also a clear division between (1) professional observatories, which are much more expensive, more difficult to robotize, and purely intended for research, and (2) amateur ones, with more modest equipment, more easily robotized, and intended more for education and dissemination. In any case, both generally share a common structure, which includes telescope, focuser, weather station, CCD and all-sky cameras, uninterrupted power supply (UPS), computers, and lights, all being protected by a motorized dome.

The robotic observatories are ideal environments for scientific research, but also for astronomy education and citizen science. Scientists, students, amateurs, and citizens, in general, can access these infrastructures through the Internet. An extensive study that was published in 2018 [1] provides the full background over a period of full background on the use of robotic telescopes in education, with numerous examples and citations. The world of astronomy fans is very exciting too and there are many published experiences [2] where amateurs describe, step-by-step, how they robotize their own observatories, both from the point of view of hardware and software. Not only that, but the authors in [3], through a review of the ideas for carrying out citizen science in Astronomy, emphasize the importance of making widely available amateur observatories for the observation of certain phenomena as compared to professional ones.

These unmanned observatories tend to be not only robotic, but autonomous or also called smart, which means that they are capable of automatically managing themselves according to their equipment status and weather conditions. They are made up of several subsystems, as described by [4], each with different features, and they require control and synchronization software for all of them to work in a coordinated, autonomous, and reliable way.

The authors of this article have worked for years on the robotization of telescopes by means of open-source software, and they have their own telescope at the Universidad Politecnica de Madrid: the Francisco Sanchez Observatory (OFS, from the acronym in Spanish). This is a unique astronomical infrastructure in a context of a university specializing in technological and engineering degrees, and it has been running since 2009. The authors have used this observatory as an experimental framework for their research and as an e-infraestructure for Astronomy outreach [5]. Since 2011, the OFS belongs to GLORIA (GLObal Robotic-telescopes Array for e-Science), a worldwide network of 17 robotic telescopes that cover four continents and both the northern and southern hemispheres [6]. GLORIA has developed open standard software that is capable of integrating and tele-operating any robotic telescopes into a network and, hence, ensuring that there is always a suitable place on the globe to virtually observe astronomical phenomena.

Today, most telescopes are focused on observing the night sky, and a few are used to study the Sun. These require a specified Hydrogen-Alpha filter that is integrated into their own structure. This is the case in GLORIA, where only one of the 17 telescopes is solar. The OFS also has a solar telescope, held by a clamp over the night-time 10-inch telescope, both being supported on an equatorial mount on the top of the pier. This configuration has made possible the design of different educational experiments, both solar and nocturnal [5]. With the original configuration, the combination of both telescopes required manual intervention to protect the night-time telescope with an ordinary cover during the day and uncover it at the beginning of the night. These covers are used to protect the CCD sensors and the optics from dust and sunlight, normally when a telescope is not used for a long time. There are some basic commercial accessories, both flexible and rigid covers, which are manually fitted over the tube aperture in front of the objective lens. There are other covers, robotized in this case, the so-called Flip-Flat, which are specific to those who work in astroimaging and photometric. They employ advanced electroluminescent lamp panel technology to provide a uniform source of illumination for the production of high quality flat field frames. Furthermore, they offer a Windows interface to control them, opening and closing the Flip-Flat and turning on the light source. Their prices range from 400 dollars, depending on the diameter of the tube, as published on the website of one of its main manufacturers, Alnitak.

In Astronomy, there is a wide variety of devices, brands, and programs. In most cases, the Pro-Am population work with commercial solutions under Windows accessible through Remote Desktop Applications in order to control their robotic observatories, or even develop their own systems, combining usually programming languages (Python, Java or C++) and Linux systems, like the networks of robotic telescopes BOOTES [7], MASTER [8], and LCOGT [9], or individual telescopes, such as DEMONEXT [10], LWT [11], MONET [12], and STELLA [13], among others.

Easy access and affordable prices for electronic components and microcontrollers, among other things, are leading to the emergence of increasingly Internet-connected devices, known as the Internet of Things (IoT) [14], which are increasingly publishing openly their designs in both hardware and software. The benefits of free/libre open-source software and hardware are described in [15] and its application has spread to more fields than computer engineering. Recently, there has been a lot of examples published [16,17,18,19].

This paper describes, in detail, an open-design smart cover for a robotic telescope in order to achieve continuous day-and-night operation in the same observatory with two telescopes, one solar and the other for nighttime. The mechanism is totally original, which minimizes the space that it occupies and being as minimally invasive to the telescope as possible. The cover has been built using low-cost conventional components and a 3-D printer and PCB milling machine. The design includes both mechanical and electronic parts, as well as the source code that is needed to program the operation. The system can also be adapted digitally, so as to customize the cover in diameter size and manufactured in any of the emergent Fabrication Laboratories (known as Fab Labs) or makerspaces at home. All of the schemes are available from a GitHub (Smart Cover-GitHub: https://github.com/cslab-upm/smart-cover (accessed on 4 February 2021)) repository, Instructables platform (Smart Cover-Instructables: https://www.instructables.com/Smart-Cover-of-a-Night-time-Telescope-for-Day-time/ (accessed on 4 February 2021)) and Zenodo (Smart Cover-Zenodo: https://zenodo.org/communities/cslab-upm/ (accessed on 4 February 2021)), created to open the design.

The rest of the article is divided into four sections, as follows. Section 2 describes the observatory where the smart cover has been conceived. Section 3 presents the complete mechatronic design of the cover; Section 4 summarizes the main results of the article; and, Section 5 gathers the main conclusions of the system and future works.

## 2. Context and Motivation

**Figure 1:**
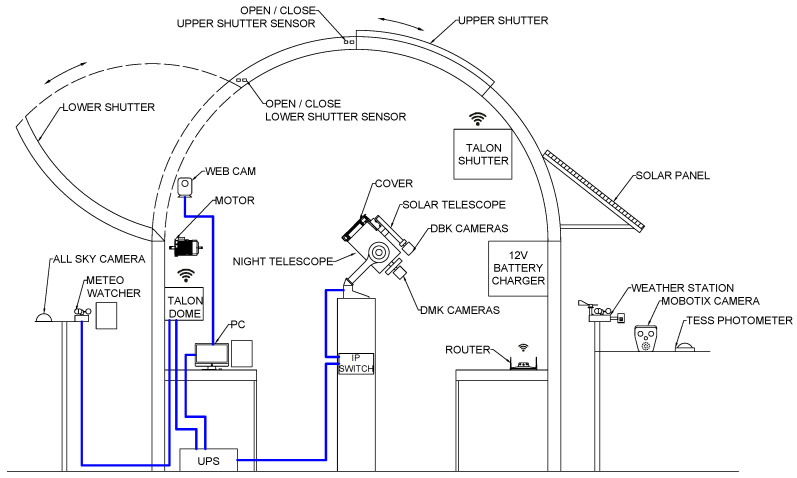
Diagram of the OFS’s (Francisco Sanchez Observatory, from the acronym in Spanish) components.

In this section, a description of the observatory in which the smart cover will be installed is given. The OFS consists of a traditional dome with a semi-spherical structure. The upper shutter slides upwards and the lower shutter opens laterally, as shown in Figure 1. The OFS is an integrated system, which is equipped with different components, both inside and outside the dome.

The dome is completely motorized and it is controlled through Talon6 (Webpage of Talon6: http://observatoriosspag.es/Whats_TALON6.htm (accessed on 4 February 2021)) system. Talon6 is specially used in remote domes and roll-off-roofs observatories, providing both manual and automated control. In case of a problem in the observatory (loss of Internet, power failure, unsuitable weather conditions, etc.), Talon6 automatically closes the shutter without human intervention. Talon6 uses an ASCOM compatible driver that can be controlled from a computer, allowing for the opening and closing of the shutter automatically at the beginning and end of a photo session and accurately positioning the dome at a particular point in synchronisation with the mount.

The components inside the dome are:

- Night telescope: it is a Meade LX200GPS with 8” (20.32 cm) on a computerized equatorial mount. Communication with the telescope is done through the serial port, following the LX200 protocol provided by the manufacturer.
- Solar telescope: it is a Coronado PST (Personal Solar Telescope) with 40 mm opening and a focal length of 400 mm. Thanks to this telescope, the Sun can be observed in the H-alpha band, making it possible to distinguish the protuberances, wails, granulation, and other details of the Sun. This telescope has been used previously to take the Sun images for calculating the solar activity though a citizen science project, as described in [20].
- Computer: it includes all of the automation and observation software, and it is used as a server for remote control.
- DMK and DBK cameras: there are two astrophotography cameras connected to both telescopes. These are Imaging Source cameras, one DBK 21AU04.AS colour and one DMK 41AU02 monochrome, with 1280 × 960 pixels. These astronomical cameras have a large number of adjustable parameters, such as brightness, gain, gamma, exposure time, and region of interest, to obtain better images. They are connected to the computer via USB 2.0 and a driver has been developed to control them and make observations through a web application.
- Webcam: its objective is to constantly broadcast, in real time through the web application, the interior of the observatory to keep track of what is happening in case of a problem.
- Uninterrupted Power Supply (UPS): it is a key piece in any robotic observatory, since, in the event of any power failure, it is capable of operating independently for approximately 10 min. In that situation, the system would detect the power failure and there would be enough energy to close the shutter and the cover to avoid any damage to the equipment, to park the mount and power off the computer.
- Talon6 IP-Switch: it is an eight-relay controller that can be managed via the Internet or manually at the observatory by means of three external buttons on the controller box, which is installed on the pier. At this moment, the automatized mount and its cover are connected to this system and it is possible to turn it on and off remotely to save energy.

In addition, outside the dome, there are the following devices that complement the observatory and ensure its proper functioning:

- Meteo Watcher: this device operates autonomously, analysing the climate parameters according to the established configuration and deciding whether the observatory should remain open or not. It monitors the main relevant environmental parameters in an astronomical observatory: Cloudiness, Ambient Temperature, Relative Humidity, Dew Point, Luminosity, Rain, and Wind. Furthermore, Meteo Watcher has a relay output that is connected to the Talon6, so it would close the observatory in the event of poor weather conditions. Communication between the Meteo Watcher and computer is via Bluetooth and it allows for the data received from the different variables to be recorded and monitored.
- Weather station: a low-cost device built with Arduino and used to monitor temperature, humidity, wind speed, and rainfall.
- All-sky camera: currently being used to detect meteors and other bright objects, but it could be used to detect clouds.
- Video camera: a MOBOTIX model with integrated infrared LEDs for day and night surveillance. Its goal is that the remote observer can quickly see the dome and shutter status.
- TESS Photometer: this device includes an infrared sensor and monitors the evolution of the sky brightness. The photometer has been developed within STARS4ALL project, a collective awareness platform for promoting dark skies in Europe, funded by the EU. A full description of these photometers can be found in [21].

The goal of the work described in this paper is to develop a smart cover for the night telescope, so that it can coexist with the day-time telescope. The smart cover will be closed when the light intensity is high, protecting the night telescope from the sunlight and allowing the use of the day-time telescope.

## 3. Mechatronic Design

**Figure 2:**
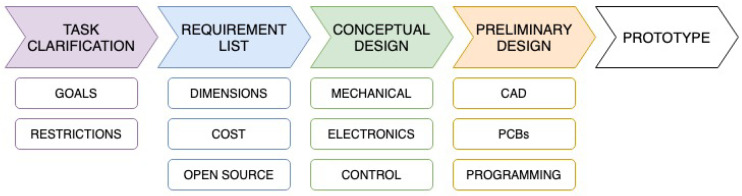
Engineering approach.

This section describes the mechatronic design of the smart cover. A systematic engineering approach has been followed in the development of the smart cover [22]. Figure 2 shows the several steps followed. The starting point is the task clarification, where the goals and restrictions are defined. It has already been carried out in Section 2. The next step that has been followed is the requirement list, where the requirements that the cover must fulfilled have been presented in terms of dimensions, cost, and open source initiatives. They are listed in Section 3.1. The next task is the conceptual and preliminary design, an iterative process that leads to a first prototype. It is described in Section 3.2 (mechanical design and CAD drawings), Section 3.3 (parameterized design), Section 3.4 (electronic design and PCB design), and Section 3.5 (control algorithms and programming). The last step is the first prototype that is described in Section 4.

### 3.1. Requirements

**Figure 3:**
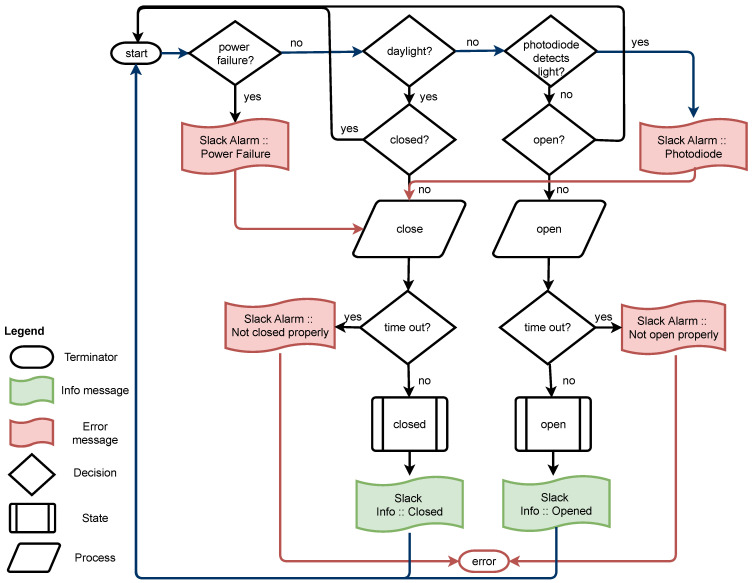
Flowchart of cover operation.

The operation of the device is managed by a controller unit, which receives the readings from the sensors and decides how to act based on certain control variables: geographic coordinates, time, light intensity, and voltage in the electrical network. The cover needs to know the time precisely in order to work: closed during the day and open at night. The system calculates the time of sunrise and sunset, depending on the latitude and longitude where the observatory is located. Furthermore, the controller adjusts its clock, automatically synchronizing the date and time with a public NTP timeserver, and manages all time information based on Universal Time Coordinated (UTC). The controller unit performs the following steps, as shown the flowchart presented in Figure 3:

- The system checks that there is no failure in the electrical network (power cuts, potential drops, etc.), by means of the voltage detection sensor. In the event that a failure is detected, the cover is immediately closed as a protective measure. This is possible, thanks to the UPS described in previous section.
- If there is no power failure, the system checks the time. The night telescope will always be closed during sunlight hours and it can only be opened at night.
- After checking that the time range is correct, the light intensity level is checked using a photodiode. This works as a double check: in the case of a failure obtaining time or in the photodiode measurements, the system will remain closed.

To guarantee the robustness of the system, the cover is provided with two mechanical limit sensors, one to detect full opening and the other to detect full closing. In addition, the system has a watchdog timer that initializes whenever the cover is opened. If the cover does not activate the opening limit switch within 8 s, then the cover automatically closes.

Thanks to a WiFi connection integrated in the micro controller, the cover can send messages to Slack. This communication platform, among other functionalities, is designed to manage projects in a collaborative way, allowing for users to monitor the states of sensors and system peripherals. The messages sent can vary from alerts indicating a system malfunction, such as a failure to open or a problem to activate a limit switch, to information messages indicating that the cover has been opened or closed correctly. In Figure 3, these two types of messages are shown in different colors: green for information messages and red for alarms. Thanks to this feature, the status of the system can be remotely monitored, giving us the possibility to act quickly and solve the failures before they cause other problems in the observatory.

### 3.2. Mechanical Design

**Figure 4:**
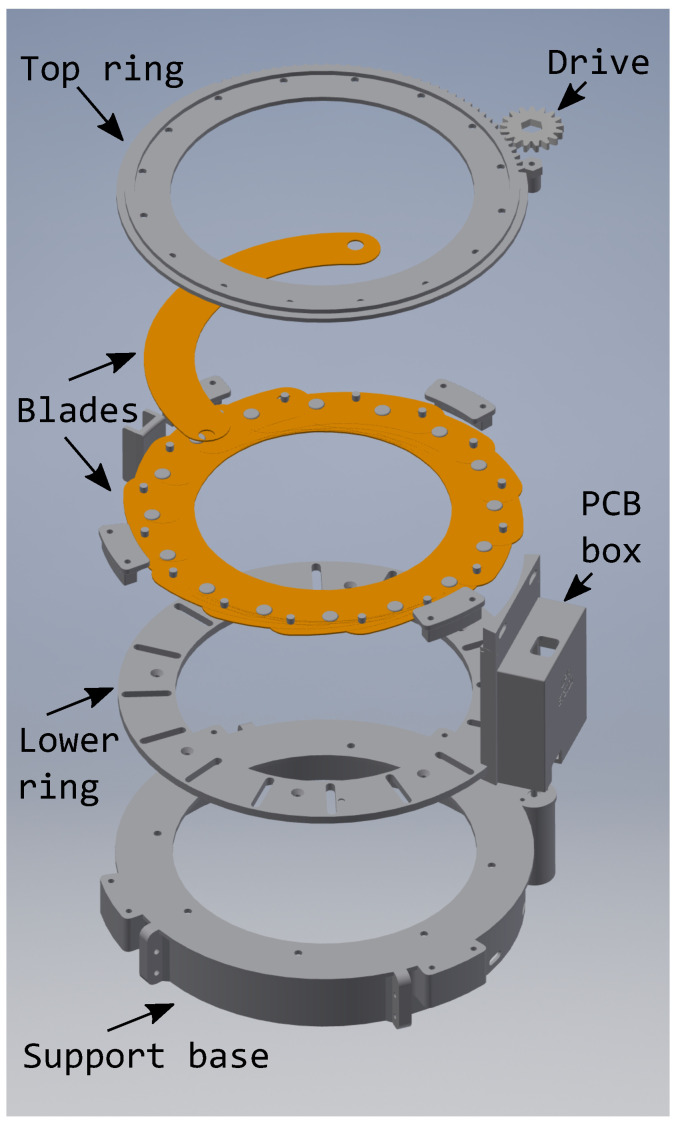
Exploded view and assembly drawing.

**Figure 5:**
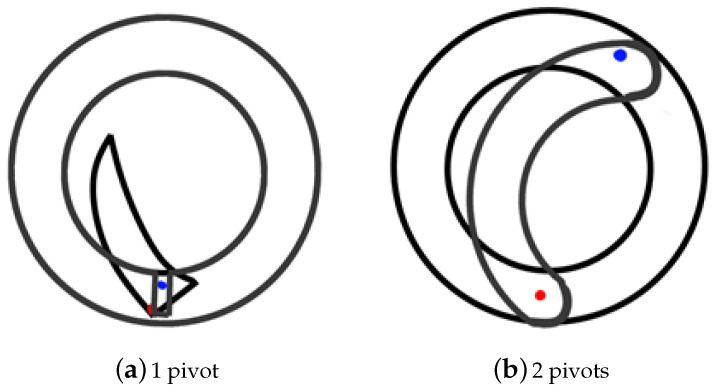
Different iris models.

The three-dimensional (3D) mechanical design (Mechanical design DOI: 10.5281/zenodo.4401783) has been carried out in Autodesk Inventor. Figure 4 shows the exploded view and assembly drawing. The mechanical design is based on an iris-type mechanism, which is similar to the one used in some cameras. Two models were considered for the iris: the first one with small thickness blades pivoting on a single point, as shown in Figure 5a, and the second one with small thickness blades as well, but, in this case, pivoting on two points, as shown in Figure 5b. The second model requires a much larger turning radius, approximately 1/3 turn of the upper ring, due to the design geometry. The model finally chosen was the second one, given the relative ease of construction, as well as a greater rigidity of the blades when opening or closing the cover.

The mechanical parts of the smart cover will be discussed in the following sections:

#### 3.2.1. Blades

**Figure 6:**
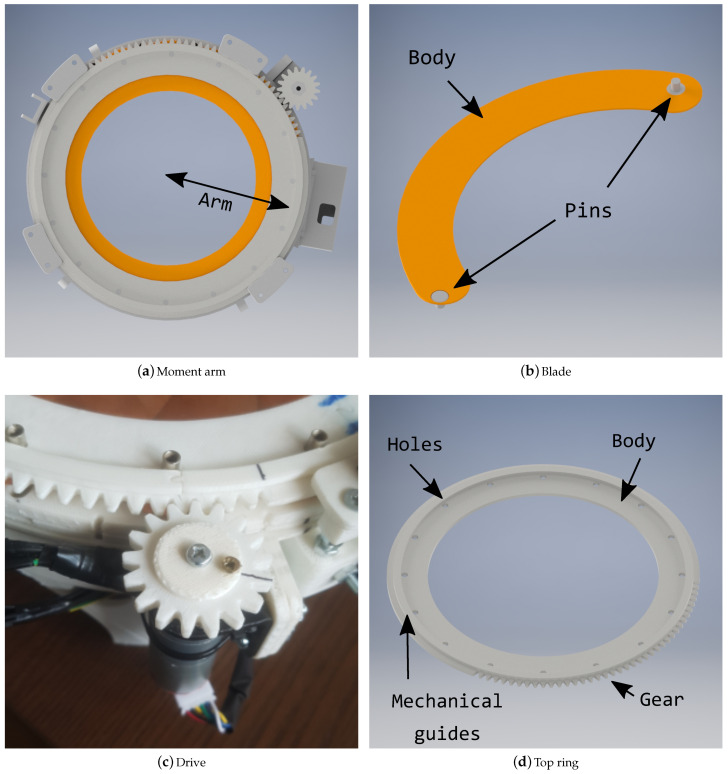
Several parts of the smart cover.

The blades are responsible for covering the lens of the telescope during sunlight. For this size of tube, sixteen 0.1 mm thick blades were made out of copper. Each blade has a pin at both ends, facing in opposite directions. The pins that are used in this prototype are button-shaped rivets, with a shaft diameter of 5 mm, as shown in Figure 6b, and they are firmly attached to the copper blade by means of super strong glue.

#### 3.2.2. DC Motor

**Figure 7:**
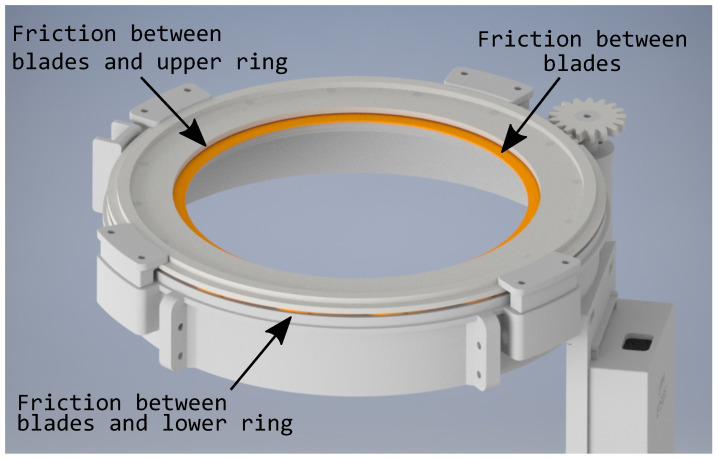
Friction forces.

In order to know the mechanical torque required by the motor, it was necessary to know all of the forces involved in the system: the weight of the elements and the friction between the different elements, which is, the friction between the blades and upper ring, the friction between the blades and the lower ring, and the friction of the blades between each other, as shown in Figure 7.

The worst case has been considered for the calculation, which is when the telescope cover is in a vertical position and all of the weight falls perpendicular to the elements. The masses of the elements have been calculated, as shown in the Table 1.

An equivalent clamping mechanism force at a mass of 50 g has been considered. This force occurs, because, when the system is opened, the blades are placed under the upper ring, as they are firmly attached to the base by this system. The blades then tend to compress each other and a force normal to the plane is generated. The total mass of the elements is 0.2636 kg, which results in a force of gravity of:

(1) $$F_{g}=M\times 9.8=2.58N$$

where *M* is the total mass.

The friction force ($F_{f}$) is calculated by multiplying the force normal to the plane ($F_{n}$) by the coefficient of friction ($f_{c}$) between the two contact surfaces (Equation (2)). The following friction coefficients have been chosen: 0.2 between the copper sheets and the upper ring and 0.1 between copper sheets.

(2) $$F_{f}=F_{n}\times f_{c}$$

The friction force for one blade is 0.31 N. The smart cover has 16 blades; consequently, the total friction force is 0.31 × 16 = 4.96 N.

When considering an actuating arm from the center of the upper ring to the center blade of 105 mm, as shown in Figure 6a, the resulting moment is: 4.96 N × 0.105 m = 0.521 N·m, which is equivalent to 5.31 Kg·cm. The maximum moment of the system is 5.31 Kg·cm, being much lower than the torque of the chosen motor (8 Kg·cm). It should be noted that this is the maximum moment to overcome at the beginning of the movement of the system, since, as the blades begin to move, the friction force between them decreases, since the area in contact between each one decreases as well as the weight applied. Furthermore, the holding force disappears, since a space is generated between the upper ring and blades.

#### 3.2.3. Top Ring

The top mobile ring is a circular crown with 16 holes for the positioning of the blade pins. The adjustment between the holes and these pins must be sliding, being smooth enough to be able to rotate. This ring consists of a machining of sprockets in part of its periphery. Because of the dimensions adopted for the small wheel, it was necessary to machine one-third of the circumference, approximately 120 teeth, as less would cause a collision between elements. These teeth were manufactured with a sprocket development module being included into Inventor. The height of the tooth is 4.33 mm, in order to ensure a good finish and rigidity through 3D printing, as can be seen in Figure 6d.

The sprocket parameters are the following ones:

- M: module = 2.
- Z: number of teeth of the sprocket = 120.
- $D_{e}$: External diameter = (Z + 2) * M.
- $D_{p}$: Primitive diameter = Z * M.
- Tooth height = 2.167 * M = 4.33 mm.

#### 3.2.4. Drive Ring

The small sprocket has been manufactured according to the space that is available due to its geometry. The result is a wheel with an external diameter of 38 mm and 17 teeth. The motor has been positioned so as not to interfere with the other elements. In the centre, a hexagonal shape has been created in order to fit the motor shaft, which has this shape, thus ensuring a secure grip. This wheel is also aligned with the upper ring, so that the teeth mesh perfectly, as can be seen in Figure 6c.

#### 3.2.5. Lower Ring

**Figure 8:**
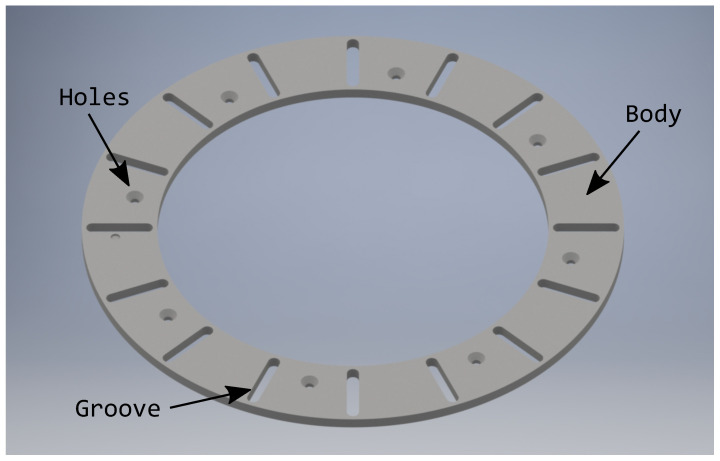
Lower ring.

The lower ring is another circular crown, but with more detail. In this case, there are 16 grooves through which the other 16 pins of the blades slide and eight holes with countersunk holes for attaching bolts to the support base. The thickness of this disc must be at least the length of the blade pin, so that there is no collision with the base, as can be seen in Figure 8.

When the upper moving ring starts to move, it pulls one end of the blade with it and the other serves as a pivot on the lower fixed ring. During the movement, the turning radius is not constant in relation to the second ring, so a second degree of freedom must be left, so that, apart from turning, it also moves over grooves in the fixed part. The simulation of this mechanism was carried out in the SAM software.

#### 3.2.6. Support Base

**Figure 9:**
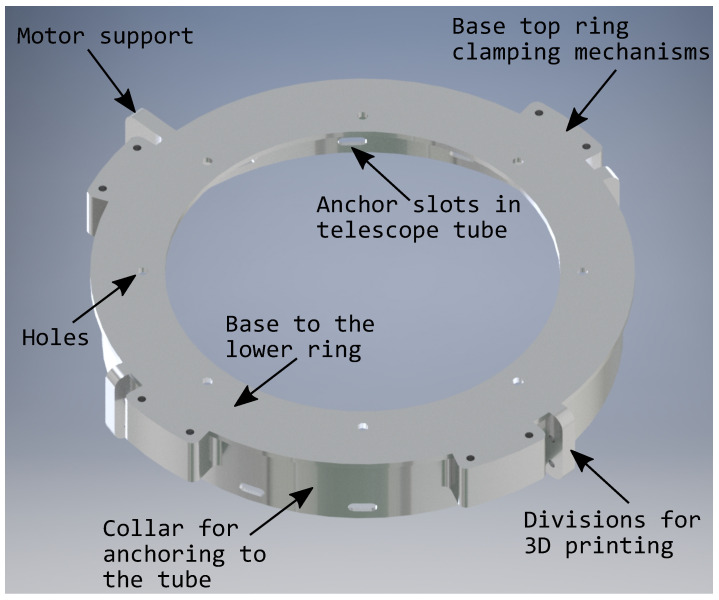
Support Base.

The support base is used to receive the other parts that make up the telescope cover and to arrange the rings and blades for its correct operation. It consists of a collar for anchoring to the telescope tube, which has seven slots to hold the base to the telescope. Above this collar is the base itself for the lower ring, where there are eight holes, which are the same as the ring itself, in order to center it. In addition, there are the bases for the upper ring fastening mechanism, which consists of extrusions that come from the collar and they are the right size for this ring to be firmly fastened with screws and other elements, as can be seen in Figure 9.

### 3.3. Parts Parameterization

**Figure 10:**
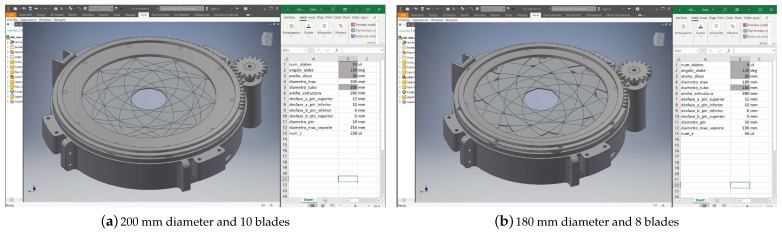
Two design parameterizations of the cover modifying diameter and blades.

**Figure 11:**
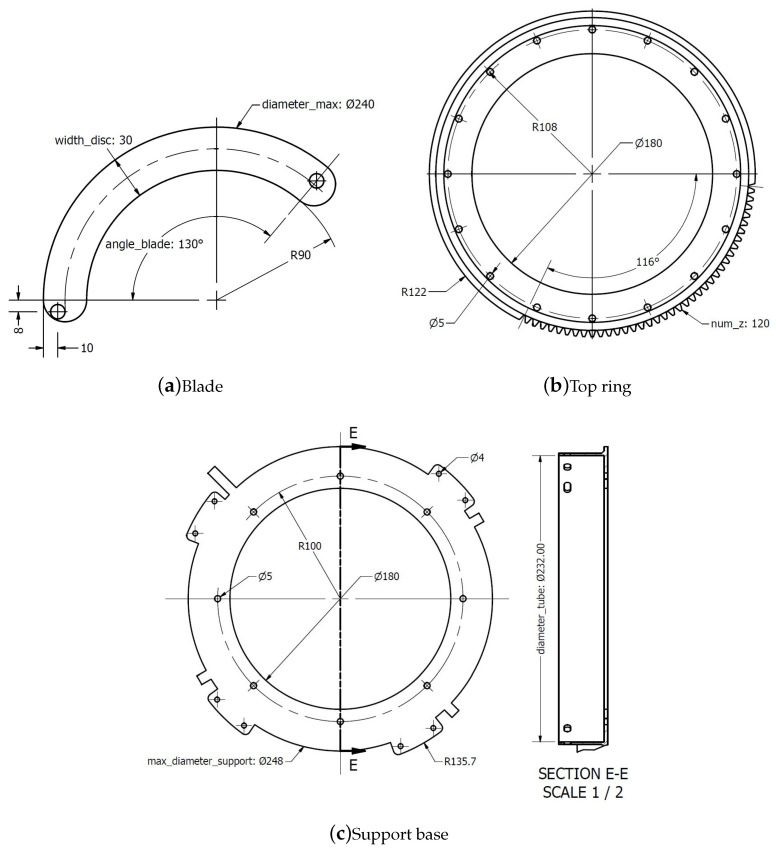
Sectional views of the cover. All dimensions in mm.

All of the parts that make up the intelligent cover are parameterized. The parameter values are in a linked excel spreadsheet. The parts can be easily resized by changing the parameter values from the excel spreadsheet. In addition, the parameters are linked, which means that changing one parameter makes the other change accordingly. An example of a parameterized part is shown in Figure 10. Table 2 shows the list of parameters. Some examples are given below and can be seen at the sectional views shown in Figure 11.

The diameter of the telescope tube is linked to the maximum diameter of the rings of the smart cover and the maximum diameter of the base of the stand. If you want to change the diameter of the tube, then it is enough to change the value of this parameter in the corresponding cell of the Excel sheet. In this way, all parts are updated at the same time.

Another parameter that can be modified is the width of the rings, both upper and lower. This conditions the amount of copper foil blades, a parameter that can also be modified at will. This way, the number of blades is up to the product developer’s consideration. This value is directly related to the number of fixing holes of the lower ring with the base. It is advisable to use a pair of blades to have exact divisions and avoid overlaps between the fixing holes and pivot holes of the blades.

Depending on the diameter of the telescope, the width of the disk or the number of blades, the other parameters may have to be slightly modified, such as the angle and offset of the blade pins and the Z parameter, which is the number of teeth on the upper ring.

The input max_diameter_support can never be greater than the input width_structure. Both of the parameters are placed in the spreadsheet to avoid collision accidents once the cover is installed.

### 3.4. Electronic Design

A specific electronic board has been developed to control the cover. The functions of this board are not only to operate the cover, but also to control its operation in order to ensure that the cover is only opened in the right conditions.

The cover, in its resting state, will always remain closed, and it will be opened if and only if the optimal conditions for its opening exist, which is: (a) to have power from the general electrical line of the observatory, (b) to be outside day-time hours, and (c) that the intensity of the light is sufficiently low so that it does not affect the lens of the telescope. Conditions (a) and (c) are quantified with two sensors selected for such work, a voltage meter and a brightness sensor. These sensors provide information to the board to determine its opening or closing. The condition (b) is verified by software, calculating the sunrise and sunset times based on latitude and longitude.

#### 3.4.1. Components

The electronic board is based on off-the-shelf components. The main components are described below.

The brain of the system is based on a ESP32 micro-controller, a cost-effective device that incorporates WiFi communications.

An integrated circuit LM293D (H-bridge) is used to drive the motor and, consequently, to open and close the cover. The speed is regulated by a PWM signal from the microcontroller. Two limit sensors have been used, connected to digital inputs of the micro-controller, in order to detect the open and close positions.

The board is powered through a voltage regulator (MP2307) that provides an adjustable output voltage between 1 V and 17 V when fed with an input voltage between 4.75 V and 23 V. It is connected to a 12 V output from the telescope. The telescope is provided with a uninterruptible power supply (UPS) that provides emergency power to a load when the main power fails. Being powered by the UPS system, the telescope can operate for around 10 min. This is time enough to close the cover, if the main power fails, to avoid that the telescope cover remains opened, and the telescope might be damaged during the day. A voltage detector FZ0430 is used in order to know when the mains power fails.

A light sensor is used to measure the light intensity. The cover is closed if there is sufficient light intensity to damage the telescope lens. A comparison module LM393 with a photodiode and a potentiometer is used to adjust the sensitivity of the measurement in order to determine this intensity.

#### 3.4.2. PCB Design

**Figure 12:**
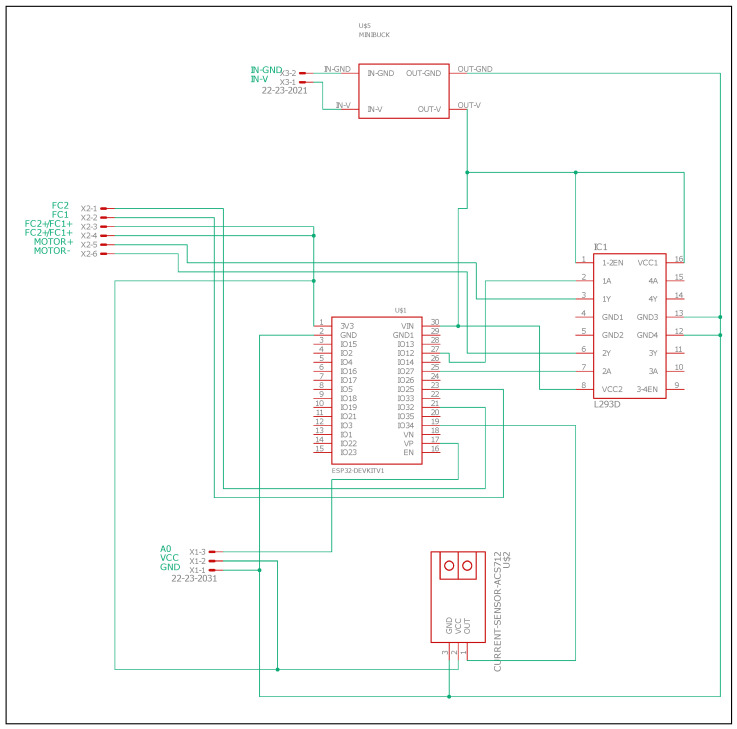
Electronic design of the main board.

**Figure 13:**
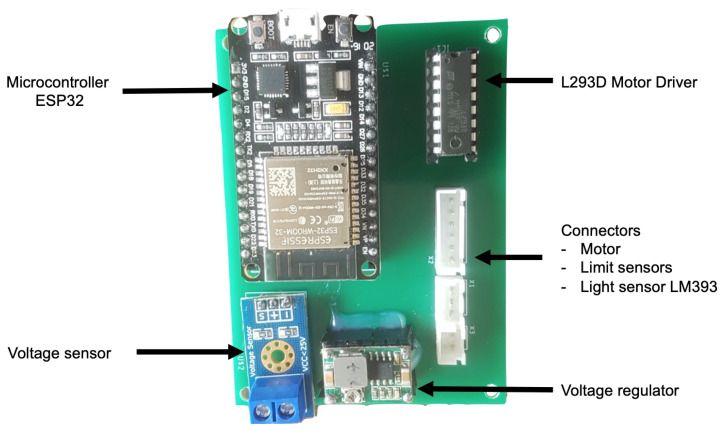
Final version of the electronic board.

The PCB (PCB DOI: 10.5281/zenodo.4401733) has been designed using Eagle. The PCB schematics can be seen in Figure 12. Additionally, the final prototype is shown in Figure 13. The PCB is enclosed in a box for protection. The external elements that have to be connected through cables are the motor, the limit sensors, and the light sensor.

#### 3.4.3. Electronics Housing

**Figure 14:**
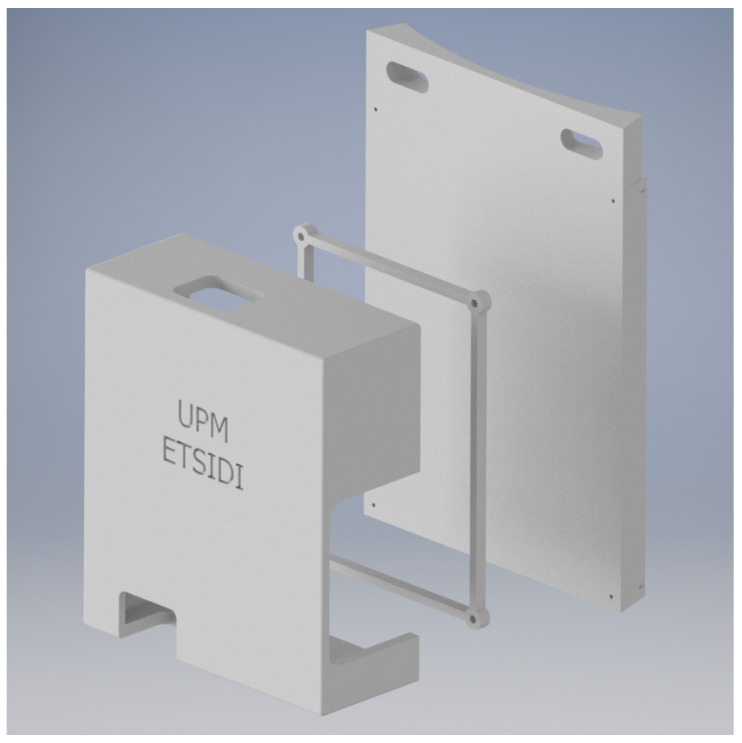
Three-dimensional (3D) printed box for the electronic board.

The electronic board box was made using 3D printing, which consists of three parts: the base, a separating element, and the cover. The base is anchored by two slots on the surface to the side of the telescope tube, in the same holes that have been used to fix the base of the smart cover. In this way, space is saved and the design is compact, so that the cables pass through as little as possible, as can be seen in Figure 14.

### 3.5. Programming

The program code (Firmware DOI: 10.5281/zenodo.4401749) was entirely made using the Arduino IDE. The ESP32 receives the information from the different sensors connected to it, and decides whether to open or close the cover, as described in Section 3.1. This program is continuously executed in order to guarantee the maximum safety of the telescope at all times in case of changes in the light intensity or power cuts that could damage the integrity of the equipment. In a summarised form, the cover will be opened if and only if it has power supply, it is outside day-time hours and the intensity of the light is sufficiently low.

As configuration parameters, the GPS coordinates of the observatory are set as properties, so that latitude and longitude are used in order to calculate the time of sunrise and sunset. For this calculation, the Arduino Dusk2Dawn library is used: given a set of a coordinates and a preferred time zone, an estimate time of apparent sunrise or sunset is returned in minutes elapsed since midnight.

The light intensity values are measured in an analogical way, so that, when a set threshold is exceeded, the cover must be closed in order not to damage the lens of the cover. In addition, the cover can only be opened if and when it is outside of daylight hours. To do this, the current time is read in seconds of the day from an NTP server, while using the NTPCliente.h library from Arduino.

In addition, when the microcontroller is ordered to open the cover, a timer is started as a watchdog. If, after N seconds, the cover is attempted to be opened and the opening limit switch does not communicate, the system is ordered to close the cover on an emergency basis and alert messages are sent to the administrators.

All the telescope actions are reported to the user through messages on the Slack platform in a workspace created for that purpose. In this way, it is possible to track whether the system has performed the opening or closing action correctly or not.

## 4. Results

**Figure 15:**
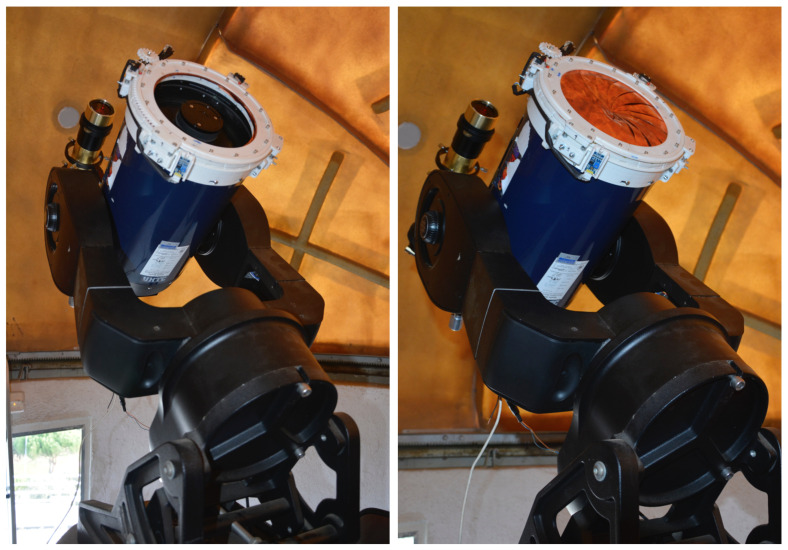
Final version of the smart cover mounted on the telescope. On the **left**: open. On the **right**: closed.

Figure 15 shows the final version of the cover, perfectly installed into the telescope. The resulting design is available under free distribution and permitted modification and derivations of it. Schematics, 3D model, and software are published in Github and Zenodo repositories, and Instructables platform, three of the most popular places to deposit and share digital artifacts.

As can be appreciated, the design is non-invasive, and it could be placed on the front of any tube. Thanks to the reduced space that it occupies, it could be installed in any type of mount, such as the altazimuth, equatorial, etc., moving freely and parking without crashing into the sides of the telescope mount.

The smart cover is customizable to any size of telescope tube. This customization process is very simple: to adjust the parameters through the excel spreadsheed, as detailed in Section 3.3.

The technical specifications for an eight-inch cover are the following ones:

- Diameter: Customizable to the size of the tube.
- Weight (for 8-inches tube): 0.380 Kg.
- Angle of movement: 120-degrees.
- Power requirements: 12 V DC.
- Time per 180-degrees: 5 s.
- Control box dimensions: 81 mm × 60 mm. This box must be held in the tube.

The maximum temperature reached inside the OFS has been 45 ºC. In this case, the PLA filement is enough for printing the 3D pieces, despite being directly exposed to the Sun.

The design that is presented in this paper is totally original, and the only similar devices that are used by similar purposes are the Flip-Flat covers exposed in Section 1. Those covers, while they might fulfil the desired function to protect the optics, do not have an intelligent behaviour and require programming, whereby the opening and closing times are indicated, leading to possible human error. In contrast, the cover that is presented here is fully autonomous and smart, based on the use of sensors and information to make its own decision on opening and closing.

**Figure 16:**
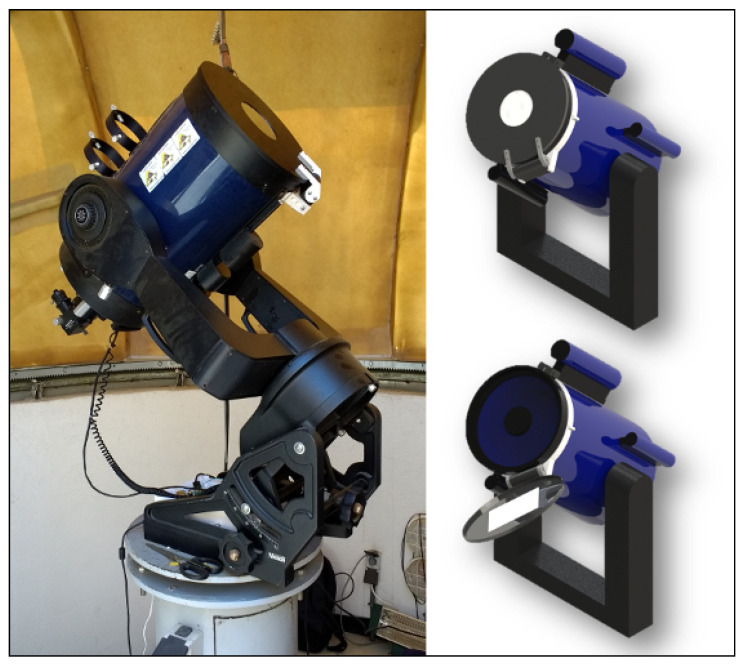
On the **left**, first prototype installed on the telescope. On the **right**, cover’s three-dimensional (3D) design in open (bottom) and closed positions (top).

The authors built a first prototype, according to the Flip-Flat design, which can be seen in Figure 16. This design, with smart-behaviour as well, consisted of a circular cover that rests on the front of the tube and keeps out light (closed position) and is opened by a mechanised arm (open position). Although the production cost was similar and the mechanical design was very simple, there were several disadvantages: (1) the use of more sensors, such as accelerometer and gyroscope, to calculate the angle of inclination of the tube, added more logic to the software; (2) the structure was more fragile against wind and possible collision with other elements, such as the mount or even people; (3) it required a brake or magnet against gravity to keep the cover in the closed position. Thus, the resulting design is much more compact and it has a more robust structure against collisions or misalignments and simpler logic.

### 4.1. Total Budget

Table 3 shows the cost of the smart cover and it is around 100 euros, a very affordable price for a smart cover for an observatory.

### 4.2. Tests

Several tests have been carried out in order to verify the proper operation of the cover, firstly in the laboratory and after that in the observatory installed in its final destination. The tests have lasted a week in both cases, covering different hours of the day and simulating the most critical hours between dawn and dusk, and vice versa.

Firstly, repeatability tests have been carried out with the following procedure: ppen the cover, wait for 15 s, and then close the cover. This test has been carried out during 15 min. under visual inspection. No single failure was detected.

Secondly, the light sensor was individually tested at different times. The cover did not open when the light intensity was above the threshold.

Finally, tests were carried out on different days ito check the amount of light passing through the cover blades in order to avoid damage to the camera sensor. To do this, a luxmeter was attached to the telescope lens, in the usual position of the CCD camera. While the telescope was pointed at the Sun, the luxmeter reached a maximum measurement of 9 lux, which is a tolerable amount of light for the camera sensors.

## 5. Conclusions

The goal of the paper was to build a low-cost smart cover that can be used in night telescopes to avoid their exposure to daylight. An innovative mechatronic design was proposed for this purpose.

For the mechanical part, an iris system was designed with small thickness copper blades. The two-point pivoting method was chosen for the blades, obtaining a greater ease of construction and greater rigidity when opening or closing them. The rest of the elements of the iris system were described in detail: DC motor, top, drive and lower rings, support base, and housing. All of these parts are parameterized, and they can be easily resized and adapted to other telescopes by changing one parameter value (i.e., external diameter). The parameters are interlinked, which means that changing one parameter makes the other change accordingly.

A cost-effective electronic board has been developed to control the cover while using off-the-shelf components. The functions of this board are not only to operate the cover, but also to control its operation to ensure that the cover is only opened in the right conditions: power source availability, night-time, and low light intensity.

The proposed design solves the problem of night-time telescopes that coexist with daytime ones, by means of an open, low-cost, lightweight, and easy to build and assemble solution. It has been tested in a real telescope for several days without any error. The final prototype weights 380 g, measures 81 mm × 60 mm, and takes 5 s to open or close the blades. The complete design is licensed under the GNU General Public License v3.0 and all of the documentation, schematics, and software are available in public repositories, like Zenodo, GitHub, and Instructables.

The scientific relevance of the proposed device is: (1) the use of sensors and IoT protocols to convert a telescope cover in a smart device; and, (2) the new parameterized design of the cover that can be adapted to other telescopes.

Future work will focus on improving exposure to high temperatures by changing the materials from PLA to aluminium or other metals, and placing a fan in the electronic board.

## Figures and Tables

**Table 1:** Components mass.

Mass of Elements				
**Item**	**Quantity**	**Description**	**Unit Mass (Kg)**	**Total Mass (Kg)**
1	1	Upper ring	0.06	0.06
2	16	Blades	0.005	0.08
3	16	Upper pins	0.0028	0.0448
4	16	Lower pins	0.0018	0.0288
5	1	Clamping force	0.05	0.05
Total mass				0.2636

**Table 2:** List of parameters of the current version for eight-inch telescope.

Parameter	Value
num_blades	16
angle_blade	130 deg
width_disc	30 mm
diameter_max	240 mm
diameter_tube	232 mm
width_structure	280 mm
max_diameter_support	248 mm
num_z	120

**Table 3:** Smart cover components cost.

Quantity	Concept	Unit Price (€)	Subtotal (€)
1	ESP32 microcontroller	9.97	9.97
1	Motor and stand	17.92	17.92
1	Copper sheets	12.99	12.99
1	Voltage sensor	1.40	1.40
1	Voltage regulator	3.51	3.51
1	LM293D	2.15	2.15
1	Light sensor	6.62	6.62
1	PLA	21.99	21.99
2	Limit sensor	0.56	1.12
1	Pivot pins	10.99	10.99
1	PCB board	13.15	13.15
Total			101.81 €

## Data Availability

Data sharing not applicable.

## Abbreviations

The following abbreviations are used in this manuscript:

GLORIA	GLobal Robotic-telescopes Array for e-Science
IoT	Internet of Things
NTP	Network Time Protocol
OFS	Francisco Sanchez Observatory
PCB	Printed Circuit Board
PLA	Polylactic Acid
Pro-Am	Professional-Amateur
UPM	Universidad Politécnica de Madrid
UPS	Uninterruptible Power Supply
UTC	Universal Time Coordinated
